# Radiographic Bone Loss and Its Relation to Patient-Specific Risk Factors, LDL Cholesterol, and Vitamin D: A Cross-Sectional Study

**DOI:** 10.3390/nu14040864

**Published:** 2022-02-18

**Authors:** Teresa Thim, Konstantin Johannes Scholz, Karl-Anton Hiller, Wolfgang Buchalla, Christian Kirschneck, Jonathan Fleiner, Johan Peter Woelber, Fabian Cieplik

**Affiliations:** 1 Department of Conservative Dentistry and Periodontology, University Hospital Regensburg, 93053 Regensburg, Germany; teresa.thim@web.de (T.T.); konstantin.scholz@ukr.de (K.J.S.); karl-anton.hiller@ukr.de (K.-A.H.); wolfgang.buchalla@ukr.de (W.B.); 2 Private Practice, 63110 Rodgau, Germany; 3 Department of Orthodontics, University Hospital Regensburg, 93053 Regensburg, Germany; christian.kirschneck@ukr.de; 4 Center of Dental Implantology, Periodontology and 3D-Imaging, 78462 Konstanz, Germany; fleiner@stricker-fleiner.de; 5 Department of Operative Dentistry and Periodontology, Faculty of Medicine, University of Freiburg, 79085 Freiburg, Germany; johan.woelber@uniklinik-freiburg.de

**Keywords:** periodontal, bone loss, LDL, vitamin D, CBCT, radiographic bone loss

## Abstract

The influence of patient-specific factors such as medical conditions, low-density lipoprotein cholesterol (LDL-C) or levels of 25-hydroxyvitamin D (25OHD) on periodontal diseases is frequently discussed in the literature. Therefore, the aim of this retrospective cross-sectional study was to evaluate potential associations between radiographic bone loss (RBL) and patient-specific risk factors, particularly LDL-C and 25OHD levels. Patients from a dental practice, who received full-mouth cone beam CTs (CBCTs) and blood-sampling in the course of implant treatment planning, were included in this study. RBL was determined at six sites per tooth from CBCT data. LDL-C and 25OHD levels were measured from venous blood samples. Other patient-specific risk factors were assessed based on anamnesis and dental charts. Statistical analysis was performed applying non-parametric procedures (Mann–Whitney U tests, error rates method). Data from 163 patients could be included in the analysis. RBL was significantly higher in male patients, older age groups, smokers, patients with high DMFT (decayed/missing/filled teeth) score, lower number of teeth, and high LDL-C levels (≥160 mg/dL). Furthermore, patients with high 25OHD levels (≥40 ng/mL) exhibited significantly less RBL. In summary, RBL was found to be associated with known patient-specific markers, particularly with age and high LDL-C levels.

## 1. Introduction

Periodontal diseases are among the most prevalent non-communicable diseases in mankind with 1.1 billion prevalent cases of severe periodontitis worldwide according to the Global Burden of Disease 2019 study [1,2]. In Germany, 51.6% of younger adults (35–44 years) and 64.6% of younger seniors (65–74 years) are affected by moderate to severe periodontal disease, as reported in the 5th German Oral Health study (DMS V) [3].

The pathogenesis of periodontal diseases is associated with the presence of subgingival biofilms and considered to be based on a host-mediated dysbiosis of the oral microbiota due to an exaggerated response of the host immune system resulting in a loss of periodontal supporting tissues [4,5,6]. General medical conditions such as diabetes mellitus or habits like smoking are associated with periodontal disease [7,8]. Recently, also metabolic disorders as well as systemic inflammation were discussed in literature as a risk of causing or accelerating periodontal bone loss [9].

One of these recently discussed conditions is hypercholesterolemia, and in particular high levels of low-density lipoprotein cholesterol (LDL-C) [10]. Hypercholesterolemia is a common phenomenon, and epidemiological data show that increased LDL-C levels (≥130 mg/dL) were prevalent in 29.4% of American adults [11]. High serum levels of LDL-C are often accompanied with a diet high in saturated or trans fats and sugar, physical inactivity, smoking, obesity, type 2 diabetes mellitus, and high blood pressure [12,13]. High LDL-C levels are also a main risk factor for atherosclerotic cardiovascular diseases [14], which represented the leading cause of death among US-Americans in 2017 [11].

Another condition, which was lately brought into focus in context with periodontitis and radiographic bone loss, is vitamin D deficiency [15,16]. Serum levels of 25-hydroxy vitamin D (25OHD), which is an intermediate product in vitamin D metabolism, lower than 20 ng/mL are defined as deficiency [17]. 25OHD deficiency is a widespread condition around the world, especially in northern regions [18], since vitamin D is mostly synthetized in the skin if exposed to sunlight, while just a little part is supplied by nutrition, e.g., by consumption of oily fish [17]. There are several health benefits known which are associated with sufficient 25OHD levels as vitamin D is important for an adequate bone mineralization and for various functions of the immune system [19,20] as well as there is a reduced risk for cardiovascular diseases [21] or cancer [22].

There are some investigations about increased LDL-C and decreased 25OHD levels within periodontitis-patients compared to healthy controls [16,23,24,25,26,27,28]. However, to the best of the authors’ knowledge, there is no study investigating on a “healthy” cross-section of daily dental patients and evaluating potential risk factors for periodontal bone loss using a highly validated method such as cone beam CTs.

Against this background, the aim of this retrospective cross-sectional study was to investigate associations between radiographic bone loss (RBL) and patient-specific general health parameters, particularly levels of LDL-C and 25OHD, in a cohort of patients treated in a private dental practice. The null-hypothesis tested was that levels of LDL-C or 25OHD, respectively, were not associated with RBL.

## 2. Materials and Methods

### 2.1. Study Design

The present study was designed as a retrospective cross-sectional study. The objective was to evaluate RBL according to patient-specific parameters including sex, age, smoking history, DMFT (decayed/missing/filled teeth) score, number of teeth, LDL-C, and 25OHD levels. Data were collected from a cohort of patients who received treatment planning for dental implants in a private practice.

The study design was approved by the internal review board of the University of Regensburg, Germany (reference: 21-2431-104; issued on 23 June 2021) in accordance with the 1964 Helsinki Declaration and its later amendments and comparable ethical standards. The study was registered at the German Clinical Trials Register (ref: DRKS00025827).

### 2.2. Patient Population

All patients from the patient pool of a private practice in Rodgau (Hessen, Germany), who had received a cone beam CT (CBCT) as well as analysis of LDL-C and 25OHD levels in the course of treatment planning for dental implants between February 2017 and October 2020 were screened for inclusion in this study. Patients were excluded if they had less than 16 teeth [29] or in case of insufficient CBCT quality. No other exclusion criteria were applied.

### 2.3. Medical and Dental History

A detailed anamnesis of the medical history and intake of medications was obtained from the dental charts and anamnesis forms, and if necessary, complemented by telephone interviews. Smoking history was recorded as pack-years (PY), which were calculated by multiplying the number of packs of cigarettes smoked per day by the number of years the person has smoked [30]. Additionally, the intake of statins or vitamin D supplementation was checked. Dental charts were checked for numbers of teeth and teeth were charted as decayed, missing, or filled (DMFT index) according to the clinical oral examination prior to the implant treatment planning, which was double-checked with the CBCT radiographs.

### 2.4. Radiographic Examination

All CBCTs were conducted with the medical indication of treatment planning for dental implants as full-mouth CBCTs (Orthophos XG 3D, Dentsply-Sirona, Bensheim, Germany). The field of view was set to 8 × 8 cm, voxel size was 160 µm, scan time was 5.1 s, the voltage was 85 kV, and the current was 7 mA.

**Figure 1:**
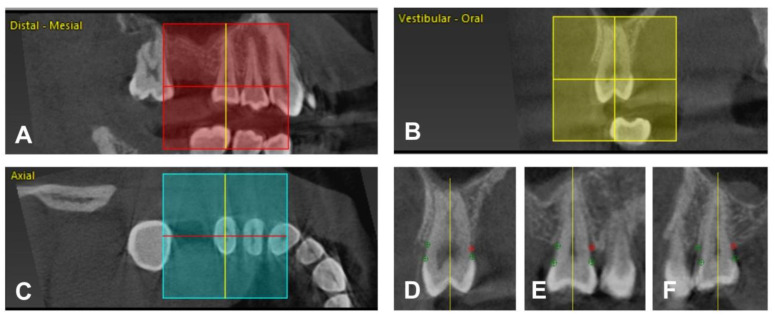
(**A**–**C**) Tooth selection and positioning. Tooth 15 in a distal-mesial (**A**), vestibular-oral (**B**), and axial (**C**) plane. The crosshair is positioned according to the longitudinal axis and CEJ or RM (**A**,**B**) and parallel to the cross-axis of the tooth (**C**). Every single tooth was selected and adjusted manually. (**D**–**F**) Setting the reference points. The yellow line marks the longitudinal axis of tooth 15. It is presented in three different cross-sections, oral/vestibular (**D**), vestibular-distal**/**oral-mesial (**E**), and vestibular-mesial/oral-distal (**F**). Twelve reference points per tooth (as depicted in green and red color) were set on the CEJ or RM and AC for each side. The red dot marks the AC oral (**D**), oral-mesial (**E**), and oral-distal (**F**). The software measures RBL by calculating the distance between the dots.

For evaluation of the radiographic bone loss (RBL), the software package CoPeriodontiX 9.9 (Dental Wings, Chemnitz, Germany) was used [31]. For each patient, all teeth except the third molars were measured. RBL was defined as distance between alveolar crest (AC) and cemento-enamel junction (CEJ) or restoration margin (RM) in cases of teeth with restorations (e.g., crowns) on six sites per tooth (mesio-buccal, buccal, disto-buccal, mesio-oral, oral, disto-oral). For measurement of RBL, every single tooth was manually positioned three-dimensionally according to its longitudinal axis and its CEJ or RM. After adjusting, CEJ or RM and AC had to be marked on six aspects of the tooth so that RBL could be calculated by the program. Figure 1 shows the workflow for determining RBL in the program package.

All CBCTs were examined by one examiner (TT), who had been extensively trained by an expert (JF). For validation of the accuracy of the RBL measurements, ten randomly chosen CBCTs were re-evaluated and differences between the first and second measurement were assessed for intra-examiner agreement.

### 2.5. LDL-C and 25OHD Levels

All evaluations of serum LDL-C and 25OHD were conducted in the course of treatment planning for dental implants. Two venous blood samples (2 mL each) were taken in a fasting state by a trained examiner from the basilic vein. The samples were sent to a specialized and accredited laboratory (Institut für medizinische Diagnostik, Berlin, Germany) for evaluation of serum levels of LDL-C and 25OHD. LDL-C levels were determined by enzymatic tests and the physical unit was mg/dL. For evaluation of 25OHD, electrochemiluminescence immunoassay (ECLIA) was conducted and 25OHD levels were measured in ng/mL. All LDL-C and 25OHD data were retrieved retrospectively from the dental charts.

### 2.6. Data Analysis

The maximum of the six RBL values per tooth was determined as the descriptive value for each tooth. The median of these maxima over all existing teeth of a patient except third molars was used as the RBL value of a patient for analysis. From all patients, medians including first and third quartiles from RBL values were calculated.

Patients were categorized, as follows: Three age groups were formed (≤44 years; 45–59 years; ≥60 years). Smoking history was differentiated in “non-smokers”, “smokers with ≤15 PY”, and “smokers with ≥16 PY”. For DMFT score and number of teeth, the patients were divided in four groups (≤11; 12–18; 19–23; ≥24) or two groups (≤24; ≥25), respectively. LDL-C levels were subdivided as “optimal” LDL-C (≤99 mg/dL), “near optimal” LDL-C (100–129 mg/dL), “borderline high” LDL-C (130–159 mg/dL) and “high” LDL-C (≥160 mg/dL) according to the U.S. National Cholesterol Education Program [32]. 25OHD levels were categorized as “deficiency” (≤19 ng/mL), “insufficiency” (20–29 ng/mL), “sufficiency” (30–39 ng/mL), and “optimal” (≥40 ng/mL) [17,19].

For analysis of RBL categorized to the different LDL-C groups, patients reporting intake of statins were excluded (*n* = 5). Accordingly, patients who reported supplementing vitamin D were excluded for RBL analyses categorized to the different 25OHD groups (*n* = 46).

Differences among experimental groups for matching parameters were evaluated statistically using non-parametric Mann-Whitney U tests on a significance level of α = 0.05. For evaluation of a general influence of a given parameter on all groups, the level of significance was adjusted to α*(k) = 1 − (1 − α)^1/k^ (k = number of pairwise tests) according to the error rates method, yielding an α*(3) = 0.01695423 and an α*(6) = 0.00851244 for three or six pairwise tests (i.e., three or four categorized groups), respectively [33]. All statistical analyses were performed using SPSS for Windows, version 26 (SPSS Inc., Chicago, IL, USA).

RBL, age, and LDL-C or 25OHD levels, respectively, were put into a three-dimensional curve-fitting model and depicted accordingly. TableCurve 3D automated surface fitting analysis software (SYSTAT Software Inc., Systat Software Inc, San Jose, CA, USA; version 4.0) was used to find equations to describe the three-dimensional empirical data.

## 3. Results

### 3.1. Patient Population

CBCTs, LDL-C, and 25OHD levels were available from 178 patients. Twelve patients with less than 16 teeth were excluded. Three patients were excluded because quality of CBCT was too poor for further analysis (extensive artifacts due to metal-based restorations). A total of 163 patients could be included in this study.

**Figure 2:**
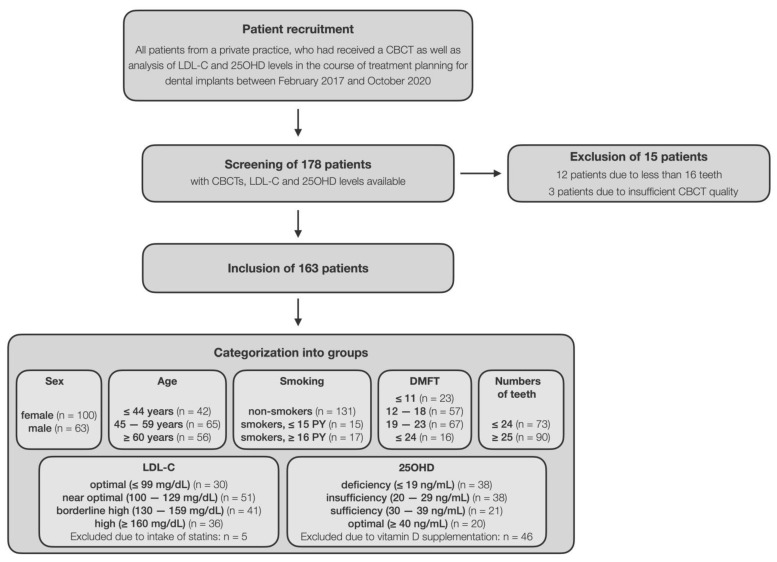
The chart depicts the flow of patients through the stages of this study.

The patient cohort comprised 100 females (61.3%) and 63 males (38.7%), the median (first; third quartile) age was 53 (44; 62) years and 80.4% were non-smokers. All patients exhibited in median (first; third quartile) 25 (22; 27) teeth, a DMFT score of 19 (14; 22), LDL-C level of 127 (107; 156) mg/dL, and 25OHD level of 29 (20; 43) ng/mL (Table 1). Median (first; third quartile) period of time between the CBCT and taking of the blood samples was 15 (1; 50) days. Figure 2 shows the flow of patients through the stages of this study.

For evaluation of the general influence of age on a given parameter, α was adjusted according to the error rates method to α*(3) = 0.01695243. *p*-value: pairwise significant difference (*p* ≤ 0.05); —: no pairwise significant difference; ^§^ significant influence of age groups on the respective parameter according to the error rates method.

Table 1 shows DMFT score, number of teeth, LDL-C and 25OHD categorized according to the distinct age groups. According to the error rates method, all age groups showed statistically significant differences with regard to DMFT score, number of teeth, and LDL-C. DMFT score and LDL-C were significantly higher, and number of teeth was significantly lower in older age groups as compared to younger age groups. For 25OHD, there were no statistically significant differences among the age groups.

### 3.2. RBL

**Figure 3:**
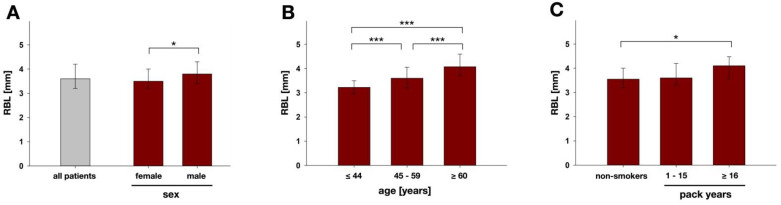
Results of RBL analysis for (**A**) all patients (*n* = 163) and divided into male (*n* = 63) and female (*n* = 100) and according to (**B**) the different age-groups (≤44 y, *n*= 42; 45–59 y, *n* = 65; ≥60 y, *n* = 56) and (**C**) smoking groups (non-smokers, *n* = 131; 1–15 py, *n* = 15; ≥16 py, *n* = 17). Results are depicted as medians, first and third quartiles and asterisks depict statistically significant differences between the groups. * marks significant differences with *p* ≤ 0.05; *** marks significant differences with *p* ≤ 0.001.

The RBL validation measurements revealed a median (first; third quartile) difference between the first and second measurements of −0.1 (−0.3; 0.1) mm, thus showing sufficient intra-examiner accuracy. Median (first; third quartile) RBL was 3.6 (3.2; 4.2) mm for all patients (Figure 3A). Females had significantly smaller median RBL (3.5 mm) than males (3.8 mm; *p* = 0.026; Figure 3A).

Patients ≤ 44 years showed significantly smaller median RBL (3.2 mm) than patients between 45 and 59 years (3.6 mm) and patients ≥ 60 years (4.1 mm). The differences in RBL were statistically significant between all age groups (*p* = 0.000 in all cases; Figure 3B) and accordingly, there was a general influence of the parameter age on RBL according to the error rates method.

Smokers reporting a smoking history of ≥16 PY had significantly higher median RBL values (4.1 mm) than non-smokers (3.6 mm; *p* = 0.029; Figure 3C). There were no significant differences between the two smoking groups or between the non-smokers and the smoking group with 1–15 PY.

**Figure 4:**
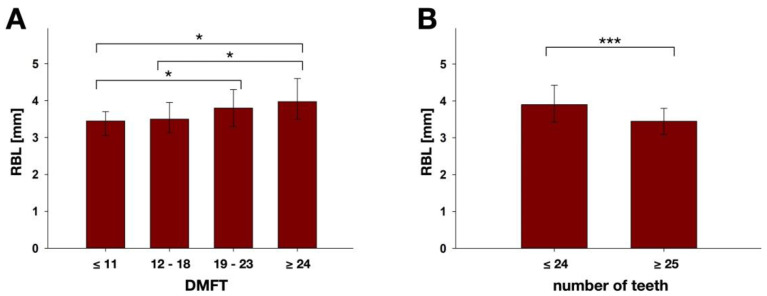
Results of RBL analysis according to (**A**) the different DMFT score groups (≤11, *n* = 23; 12–18, *n* = 57; 19–23, *n* = 67; ≥24, *n* = 16) and (**B**) number of teeth (≤24, *n* = 73; ≥25, *n* = 90). Results are depicted as medians, first and third quartiles and asterisks depict statistically significant differences between the groups. * marks significant differences with *p* ≤ 0.05; *** marks significant differences with *p* ≤ 0.001.

DMFT score analysis showed that RBL increased with DMFT (Figure 4A). Patients with DMFT score ≤ 11 showed significantly smaller median RBL (3.5 mm) as compared to patients with DMFT score between 19 and 23 (3.8 mm; *p* = 0.036) and patients with DMFT score ≥ 24 (4.0 mm; *p* = 0.019). Likewise, patients with DMFT score between 12 and 18 showed significantly smaller median RBL (3.5 mm) than those with DMFT score ≥ 24 (*p* = 0.020). It was also found that patients with 24 teeth and less had significantly higher median RBL (3.9 mm) than the ones with ≥25 teeth (3.5 mm; *p* = 0.000), as shown in Figure 4B.

**Figure 5:**
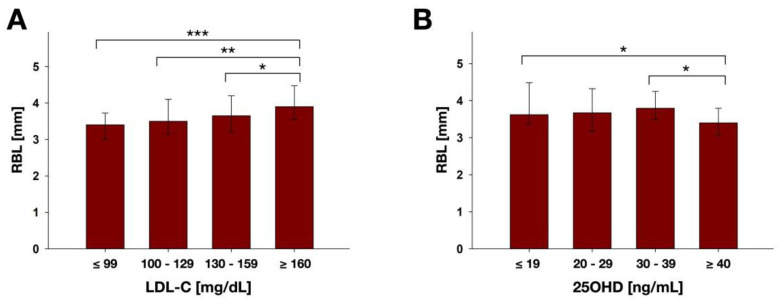
Results of RBL analysis according to the different (**A**) LDL-C groups (≤99, *n* = 30; 100–129, *n* = 51; 130–159, *n* = 41; ≥160, *n* = 36) and (**B**) 25OHD groups (≤19, *n* = 38; 20–29, *n* = 38; 30–39, *n* = 21; ≥40, *n* = 20). Results are depicted as medians, first and third quartiles and asterisks depict statistically significant differences between the groups. * marks significant differences with *p* ≤ 0.05; ** marks significant differences with *p* ≤ 0.01; *** marks significant differences with *p* ≤ 0.001.

Figure 5 shows RBL according to LDL-C (Figure 5A) and 25OHD groups (Figure 5B). Patients with high LDL-C (≥160 mg/dL) showed significantly higher median RBL (3.9 mm) than those with optimal (≤99 mg/dL; 3.4 mm; *p* = 0.000), near optimal (100–129 mg/dL; 3.5 mm; *p* = 0.009) and borderline high LDL-C (130–159 mg/dL; 3.7 mm; *p* = 0.033). Accordingly, the error rates method revealed a general influence of the parameter LDL-C on RBL. Patients with optimal 25OHD (≥40 ng/mL) showed significantly lower median RBL (3.4 mm) than those with deficient (≤19 ng/mL; 3.6 mm; *p* = 0.029) and sufficient 25OHD (30–39 ng/mL; 3.8 mm; *p* = 0.031). No general influence of the parameter 25OHD on RBL was detected by the error rates method.

**Figure 6:**
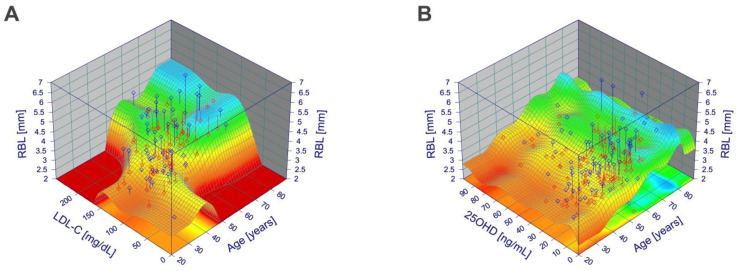
Influence of age and LDL-C or 25OHD levels, respectively, on RBL as fitted three-dimensional curves. (**A**) Influence of age and LDL-C level on RBL as fitted three-dimensional curve (Cosine Series Bivariate Order 5; r^2^ = 0.44). (**B**) Influence of age and 25OHD level on RBL as fitted three-dimensional curve (Fourier Series Simple Order 2 × 4; r^2^ = 0.37).

RBL, age, and LDL-C or 25OHD levels, respectively, were put into a three-dimensional curve-fitting model and depicted accordingly. When depicting age and LDL-C, Figure 6A shows an irreducible influence of both parameters on RBL. When depicting age and 25OHD, Figure 6B shows that RBL is mainly influenced by age but not by 25OHD levels.

## 4. Discussion

The aim of the present study was to investigate potential associations between RBL and patient-specific parameters like sex, age, smoking history, DMFT score, number of teeth, LDL-C, and 25OHD in a cohort of patients, who received treatment planning for dental implants. Focusing on RBL as reference value allowed evaluation of the accumulated history of periodontal destruction and reflected a longer period of time, regardless of the current state of clinical periodontal health.

The data of our study showed that there were significantly higher RBLs in males and in the older age groups, which is in line with other studies. Helmi et al. also evaluated radiographic alveolar bone loss in a cohort-study and revealed significant higher RBLs in men and older age groups [34]. Eke et al. found a higher prevalence of periodontal disease in men as well as an increasing prevalence of periodontitis in the older age groups [35]. Aging is accompanied with modifications of the host immune response, which leads to greater susceptibility to infections and autoimmunity [36]. The higher RBL in males may be explained since men may be less attentive to their (oral) health and consequently may exhibit worse oral hygiene levels, leading to higher RBLs [37]. Furthermore, the immune response is different in men and women, whereby men show higher levels of pro-inflammatory cytokines during infections [37,38].

Furthermore, RBL was also found to be significantly higher in smokers with ≥16 PY compared to non-smokers. A similar outcome could be found in other studies, which had measured radiographic alveolar bone loss [34,39]. Smoking is known as a risk factor for onset and progression of periodontitis [40,41] and has also been included as grade modifier in the 2018 classification of periodontal diseases [42]. Smoking is known to impair the host response to the dental plaque biofilm and to be linked to increased levels of potentially destructive inflammatory cytokines and enzymes [43]. Furthermore, smoking diminishes the reparative capacity of periodontal cells, including fibroblasts, osteoblasts and cementoblasts, thus potentially resulting in a higher RBL in smokers [43].

The patient cohort investigated in the present study exhibited a median DMFT score of 19 with a median number of 25 teeth per patient, which clearly outnumbers the results reported in the fifth German Oral Health study for the respective age groups (mean DMFT score of 11.2 or 17.7 for age groups 35–44 or 65–74, respectively) [3]. The reason might be the fact that the investigated cohort were seeking for treatment with dental implants. The older age groups showed significantly higher DMFT score, significantly lower number of teeth, and significantly higher RBL than patients from the other groups. To the best of the authors’ knowledge, there has been no other study investigating RBL and DMFT score or numbers of teeth. Levin et al. evaluated the DMFT score and number of missing teeth in periodontitis patients compared to healthy controls [44]. They did not find statistically significant differences regarding DMFT scores but did find a significantly higher number of missing teeth in periodontitis patients. Strauss et al. and Mattila et al. investigated the co-occurrence of periodontitis and caries and found significantly higher numbers of decayed teeth in patients with periodontitis [45,46]. Tooth loss represents the end stage of oral diseases such as periodontitis or caries, thus representing an objective marker for the accumulated inflammatory burden of oral disease. Therefore, there may be common risk factors for caries, periodontitis, and tooth loss such as nutrition, limitations in oral hygiene, and not seeking dental treatment [45].

Pre-conditions such as high LDL-C levels or insufficient levels of 25OHD may be linked to higher RBL [10,15]. Median LDL-C was 127 mg/dL in the patient cohort. According to Virani et al. mean LDL-C among American adults was 112.1 mg/dL and prevalence of LDL-C levels ≥ 130 mg/dL was 29.4% [11]. In our study, 47.9% were found to have LDL-C levels ≥ 130 mg/dL. Thus, the investigated patient cohort had slightly higher values of LDL-C than the average population. As found by Waskiewicz et al., patients suffering from tooth loss and thus seeking for treatment with dental implants might have higher LDL-C values [47]. Significantly higher LDL-C levels were found in the older patient groups which is in line with the literature [48,49], and may be due to an age-associated loss of hepatic LDL receptors, higher body-mass index, larger waist circumference and lower sex hormone levels [49].

LDL-C was found to have a significant influence on RBL according to the error rates method and the high LDL-C group (≥160 mg/dL) exhibited significantly higher RBL than all other groups in pairwise comparisons. Due to the general influence of age groups on LDL-C as well as RBL according to the error rates method, it cannot be clarified entirely from the data of this study which of both parameters had the bigger influence on RBL (see Figure 6A). However, there are a few more studies, which reported significant associations between high LDL-C and periodontitis when investigating clinical parameters [23,24,25,26]. Furthermore, a meta-analysis and meta-regression concluded that periodontitis patients had significantly higher levels of LDL-C [10]. Conversely, Monteiro et al. and Saxlin et al. did not find any significant differences between periodontitis patients and healthy control patients regarding levels of LDL-C [50,51]. Potential associations between LDL-C and periodontal status can be discussed in two ways. The presence of a periodontal infection negatively affects serum lipid levels by an altered immune cell function which leads to a dysregulation of the lipid metabolism [23,52]. On the other hand, high LDL-C levels lead to an increase in periodontal destruction because of an activation of osteoclasts and inhibition of osteoblasts [53] and by the release of pro-inflammatory cytokines [8,54]. Furthermore, higher LDL-C values can be interpreted as a marker of disease-promoting lifestyles, which primarily lead to a higher periodontal inflammation [12,55]. There are also common gen polymorphisms, which are risk factors for both diseases, periodontal disease and hyperlipidemia [56].

The median 25OHD level was 29 ng/mL in the present study, and 23.9% of all patients showed vitamin D deficiency (≤19 ng/mL). Another German investigation found median 25OHD of 44.9 mmol/L, which correspond to 18 ng/mL, and 57.3% of 3,917 subjects were found to be deficient of 25OHD [57]. We found no notable difference regarding 25OHD levels between the different age groups, which is in line with the literature [58,59].

RBL was found to be significantly higher in patients with “deficient” 25OHD levels found as compared to patients with “optimal” (≥40 ng/mL) 25OHD levels. These results are in line with a recently published systematic review and meta-analysis concluding that periodontitis is associated with lower 25OHD levels [15]. This concurs with the known bone-protective effect of higher 25OHD levels, which have been shown to decrease the ratio of RANKL to OPG expression by periodontal ligament fibroblasts controlling osteoclastogenesis [60]. Ketharanathan et al. focused on radiographic bone loss in their cohort study to investigate the impact of 25OHD levels in periodontitis patients. They found that patients with periodontal disease comprised higher radiographic alveolar bone loss (as measured on bitewing radiographs) and lower 25OHD which corresponds to our findings [16]. In another study, clinical attachment loss was evaluated and compared to 25OHD levels [58]. There was significantly less attachment loss in patients with high 25OHD levels, but only in the older age group (≥60 years) [58], which matches with the results from the present study, where age definitely had a higher influence on RBL than 25OHD levels, as depicted in Figure 6B. Similar results have also been shown by other studies [27,28]. Noteworthy, Perić et al. found a tendency for better healing outcomes following non-surgical periodontal therapy in patients who took vitamin D as a supplement than in patients without vitamin D supplementation [61]. There are two potential ways that vitamin D may affect the periodontal status. First, there are effects on bone mineral density especially in the elderly [62], and second, vitamin D may reduce gingival inflammation through anti-inflammatory effects on the general host immune response [15,63,64]. In addition, low salivary levels of 25OHD were found to be associated with higher levels of inflammatory biomarkers in periodontitis patients [65]. Furthermore, there is evidence that vitamin D supplementation reduces systemic inflammation and levels of pro-inflammatory salivary cytokines [66,67] and gingival bleeding [64], whereas vitamin D deficiency is supposed to be a risk factor for periodontal treatment failure [68]. It is also suggested that genetic variants of the vitamin D receptor are a biomarker for periodontitis [69].

As a potential limitation of the present study, it must be emphasized that RBL shows a history of periodontal destruction and aging, but gives no information on the current state of clinical periodontal health. Although aging is also strongly associated with RBL (as shown in Figure 6) [70], periodontal disease is considered to be the major cause for alveolar bone loss [71]. Accordingly, other studies showed that there is a reliable relationship between clinical and radiographic bone loss [72,73,74]. Clinical bone loss precedes radiographic findings six to eight months [75]. In addition, a high accuracy of CBCTs in periodontal diagnosis, especially in visualizing periodontal intra-bony and furcation defects, has been shown [76,77].

While DMFT score, number of teeth, and smoking history also reflect a longer period of time, measurement of LDL-C and 25OHD levels just reflects a current snapshot. Nevertheless, it may be assumed that the determined LDL-C and 25OHD levels are a marker for individual lifestyle and health constitution of the patients and are stable for longer periods of time, particularly due to exclusion of patients receiving “treatment” in the form of statins or vitamin D supplementation.

Although diabetes mellitus and rheumatoid arthritis are known to be associated with periodontal disease [78,79], no sub-analysis regarding those parameters was possible in the present cohort of patients since there were only two diabetes mellitus and four rheumatoid arthritis patients. The small number may be explained by the fact that only patients were included who were treated with dental implants, where diabetes and rheumatoid arthritis are known to be relative contraindications for treatment with dental implants [80,81].

## 5. Conclusions

The present study detected significant associations between RBL and patient-specific parameters like sex, smoking history, DMFT score, number of teeth, 25OHD levels, and particularly age and LDL-C. While RBL gives no information on the current state of clinical periodontal health, but reflects the cumulated burden of periodontal destruction, the outcomes of this study support similar findings of previous studies investigating clinical periodontal parameters. Future studies using RBL measurements should also include clinical periodontal parameters as well as further investigations of the association between lifestyle- and nutrition-linked conditions such as LDL-C and 25OHD levels and periodontal bone loss.

## Figures and Tables

**Table 1:** Patient characteristics (DMFT score, number of teeth, LDL-C, 25OHD). Depiction of medians (first; third quartiles) and statistically significant differences from pairwise comparisons (Mann–Whitney U tests; α = 0.05).

	All Patients	≤44 Years	45–59 Years	≥ 60 Years	≤44 vs. 45–59	≤44 vs. ≥60	45–59 vs. ≥60
DMFT score ^§^	19 (14; 22)	14.5 (11; 19)	18 (14; 20)	21 (18.3; 23)	0.025	0.000	0.000
Number of teeth ^§^	25 (22; 27)	27 (24.8; 28)	25 (22.5; 27)	23 (19.3; 25)	0.005	0.000	0.002
LDL-C ^§^ [mg/dL]	127 (107; 156)	109.5 (91.3; 127.5)	132 (113; 151.5)	146.5 (118.3; 174.3)	0.000	0.000	0.028
25OHD [ng/mL]	29 (20; 42)	27.5 (19.8; 40)	30 (19.5; 47)	30 (20.5; 39)	–	–	–

## Data Availability

All data supporting the reported results are available upon request from the corresponding author.
